# Evaluation of co-transfer of plasmid-mediated fluoroquinolone resistance genes and *bla*_NDM_ gene in *Enterobacteriaceae* causing neonatal septicaemia

**DOI:** 10.1186/s13756-019-0477-7

**Published:** 2019-02-27

**Authors:** Shravani Mitra, Suchandra Mukherjee, Sharmi Naha, Pinaki Chattopadhyay, Shanta Dutta, Sulagna Basu

**Affiliations:** 1 Division of Bacteriology, ICMR-National Institute of Cholera and Enteric Diseases, P33, CIT Road, Scheme XM, Beliaghata, Kolkata, 700010 India; 2 Department of Neonatology, Institute of Post-Graduate Medical Education & Research and SSKM Hospital, Kolkata, 700020 India

**Keywords:** Ciprofloxacin, NDM, *Enterobacteriaceae*, Neonates, PMQRs, India

## Abstract

**Background:**

The *bla*_NDM-1_ (New Delhi Metallo-β-lactamase-1) gene has disseminated around the globe. NDM-1 producers are found to co-harbour resistance genes against many antimicrobials, including fluoroquinolones. The spread of large plasmids, carrying both *bla*_NDM_ and plasmid-mediated fluoroquinolone resistance (PMQR) markers, is one of the main reasons for the failure of these essential antimicrobials.

**Methods:**

*Enterobacteriaceae* (*n* = 73) isolated from the blood of septicaemic neonates, admitted at a neonatal intensive care unit (NICU) in Kolkata, India, were identified followed by PFGE, antibiotic susceptibility testing and determination of MIC values for meropenem and ciprofloxacin. Metallo-β-lactamases and PMQRs were identified by PCR. NDM-positive isolates were studied for mutations in GyrA & ParC and for co-transmission of *bla*_NDM_ and PMQR genes (*aac(6′)-Ib-cr, qnrB, qnrS*) through conjugation or transformation. Plasmid types, integrons, plasmid addiction systems, and genetic environment of the *bla*_NDM_ gene in NDM-positive isolates and their transconjugants/ transformants were studied.

**Results:**

Isolated *Enterobacteriaceae* comprised of *Klebsiella pneumoniae* (*n* = 55), *Escherichia coli* (*n* = 16), *Enterobacter cloacae* (*n* = 1) and *Enterobacter aerogenes* (*n* = 1). The rates of ciprofloxacin (90%) and meropenem (49%) non-susceptibility were high. NDM was the only metallo-β-lactamase found in this study. NDM-1 was the predominant metallo-β-lactamase but NDM-5, NDM-7, and NDM-15 were also found. There was no significant difference in ciprofloxacin non-susceptibility (97% vs 85%) and the prevalence of PMQRs (85% vs 77%) between NDM-positive and NDM-negative isolates. Among the PMQRs, *aac(6′)-Ib-cr* was predominant followed by *qnrB1* and *qnrS1.* Twenty-nine isolates (40%) co-harboured PMQRs and *bla*_NDM_, of which 12 co-transferred PMQRs along with *bla*_NDM_ in large plasmids of IncFIIK, IncA/C, and IncN types. Eighty-two percent of NDM-positive isolates possessed GyrA and/or ParC mutations. Plasmids carrying only *bla*_NDM_ were of IncHIB-M type predominantly. Most of the isolates had IS*Aba125* in the upstream region of the *bla*_NDM_ gene.

**Conclusion:**

We hypothesize that the spread of PMQRs was independent of the spread of NDM-1 as their co-transfer was confirmed only in a few isolates. However, the co-occurrence of these genes poses a great threat to the treatment of neonates.

**Electronic supplementary material:**

The online version of this article (10.1186/s13756-019-0477-7) contains supplementary material, which is available to authorized users.

## Background

Fluoroquinolones are considered as critically important antimicrobials by the World Health Organization [1]. They are used extensively to treat gram-negative and some selective gram-positive bacteria. Quinolones (Nalidixic acid) and fluoroquinolones (Ciprofloxacin, gatifloxacin etc.) are bactericidal antimicrobials that selectively target the action of gyrase and topoisomerase IV disabling the DNA replication [2]. The classical mechanisms of fluoroquinolone resistance are the accumulation of mutations in the target enzymes and upregulation of the efflux pumps. Both these mechanisms are mutational and are passed vertically to the surviving progeny. Adding fuel to this fire are the plasmid-mediated quinolone resistance (PMQR) genes which raise greater concern because of their transmissibility. PMQRs include pentapeptide Qnr protein genes (*qnrA, qnrB, qnrS, qnrC, qnrD*) which give protection to gyrase and topoisomerase IV, fluoroquinolone modifying enzyme *aac(6′)-Ib-cr* which is a variant of the acetyltransferase of aminoglycosides, and plasmid DNA encoded efflux pumps *qepA* and *OqxAB*. Although PMQRs confer low-level resistance, they facilitate the selection of mutations in gyrase and topoisomerase genes which results in high-level resistance [3].

With the emergence of carbapenem resistance in *Enterobacteriaceae*, treatment options have been severely jeopardized. Though a number of carbapenemases (IMP, VIM, SIM, SPM, GIM, KPC, SME) have been identified in *Enterobacteriaceae*, the advent of NDM-1 has been the ‘last straw’ in this growing problem. This study focuses on *bla*_NDM-1_ instead of other carbapenemases because it is widely prevalent in India, Bangladesh, and Pakistan [4]. It is a metallo-β-lactamase that contains zinc at its active site and can hydrolyze not only carbapenems but almost all hydrolyzable β-lactams except aztreonam [4]. Apart from resistance to β-lactam antibiotics, most *bla*_NDM-1_ carrying *Enterobacteriaceae* are also resistant to a wide range of non-β-lactam antibiotics such as aminoglycosides, fluoroquinolones, sulphonamides, trimethoprim, chloramphenicol [5].

Both PMQRs and NDM-1 are present on transmissible elements and several studies have shown the presence of PMQRs with *bla*_NDM_ [5, 6]. With increasing resistance to carbapenems, and concurrent resistance to fluoroquinolones in NDM-possessing isolates, a better understanding of this association is necessary. This study focuses on fluoroquinolone non-susceptibility and prevalence of PMQRs in NDM-positive and NDM-negative *Enterobacteriaceae* isolated from cases of neonatal septicaemia. It also highlights the possibility of co-transmission of these resistance genes in single large conjugative plasmids.

In developing countries neonates are prescribed fluoroquinolones for life-threatening infections [7] and so are carbapenems [8]. A thorough evaluation of their resistance level also makes this study clinically relevant.

## Materials and methods

### Identification of strains

*Enterobacteriaceae* (*n* = 73) obtained from blood cultures of 66 septicaemic neonates (new-borns less than 28 days of life), admitted to the neonatal intensive care unit of IPGMER and SSKM Hospital, Kolkata, India, during January 2012 to June 2014, were included in this study. The isolates were identified by 5 biochemical tests which include Triple Sugar Iron test, Mannitol motility test, Simmons citrate agar test, Urease test, Indole test, and discrepancies were resolved by Vitek2 system (bioMe’rieux, Marcy l’E’toile, France). Due to unavoidable circumstances, isolates were not collected between 2012 June to 2012 December_._

### Antimicrobial susceptibility testing and determination of MIC values

The antimicrobial susceptibility testing for different antibiotic agents (piperacillin (100 μg), cefotaxime (30 μg), cefoxitin (30 μg), aztreonam (30 μg), meropenem (10 μg), ciprofloxacin (5 μg), ofloxacin (5 μg), amikacin (30 μg), gentamicin (10 μg), tigecycline (15 μg), and trimethoprim/sulfamethoxazole (1.25 μg /23.75 μg) (BD Diagnostics, Franklin Lakes, NJ, USA) was done by the Kirby-Bauer standard disk diffusion method. The MIC values (mg/L) of meropenem and ciprofloxacin were determined using Etest (AB Biodisk, Solna, Sweden). All the values were interpreted according to CLSI guidelines [9] except for tigecycline, which was interpreted according to EUCAST guidelines 2013 [10]. MIC_50_ and MIC_90_ (MIC at which 50 and 90% of the isolates were inhibited respectively) were calculated for meropenem and ciprofloxacin.

### Genotypic detection of resistance markers

PCR assays were performed on all isolates for the detection of carbapenemase genes (*bla*_NDM_, *bla*_VIM_, *bla*_IMP_, *bla*_SPM_, *bla*_GIM_, *bla*_SIM,_ *bla*_KPC,_ *bla*_OXA-48_) [8, 11–13], other β-lactamase genes (*bla*_CTX-M_, *bla*_TEM_, *bla*_SHV_, *bla*_OXA-1_) [14, 15] and PMQR genes (*qnrA, qnrB, qnrS, qnrC, qnrD*, *aac(6′)-Ib-cr, qepA, oqxA, oqxB*) [3, 16]. The *qepA* and *aac(6′)-Ib-cr* genes were analyzed by a multiplex PCR with a buffer suitable for GC rich sequences as the GC content of *qepA* gene is high (70%). The *aac(6′)-Ib-cr* was differentiated from its wild-type allele by digestion with *BtsC*I enzyme (New England Biolabs, Massachusetts) [16]. Primers used do not discard the presence of the non-ESBL variants of *bla*_TEM_ and *bla*_SHV_. As the study focuses on *bla*_NDM-1_ and PMQRs, the PCR products of *bla*_TEM_ and *bla*_SHV_ genes were not further sequenced and this remains a shortcoming of the study.

### Sequencing

All *bla*_NDM_ and *qnrB* amplified products were sequenced using primers described previously [17, 18]. *qnrS* was amplified with a pair of primers designed in this study:- qnrSF5’- TCTAGCCCTCCTTTCAACAAG-3′ and qnrSR:5′- TGAGCGTTTAAAATCACACATCA-3′. Additionally, in NDM-positive isolates, quinolone resistance determining region (QRDR) of *gyrA* and *parC* genes were sequenced [19]. Sequencing was carried out using Big-Dye terminator v3.1 cycle sequencing kit (Applied Biosystems, Foster City, USA) in an automated DNA sequencer (Applied Biosystems 3730DNA Analyzer, Perkin Elmer, USA).

### Pulsed-field gel electrophoresis (PFGE)

Genetic relatedness of the isolates was examined by PFGE in a CHEF-DRIII apparatus (Bio-Rad Laboratories, Hercules, and CA) following digestion of genomic DNA with *Xba*I enzyme (New England Biolabs, Massachusetts) according to Tenover et al. [20]. The PFGE images were processed and the dendrogram was calculated by FPQuest software v4.5 (Biorad laboratories inc, Hercules, California, USA.) using Dice coefficient and UPGMA (unweighted pair group method using arithmetic averages). Isolates having more than 95% similarity were considered identical.

### Molecular characterization of NDM-positive isolates with a focus on fluoroquinolone resistance

Transmissibility of *bla*_NDM_ was studied by conjugation experiment. In the solid mating assay, donor strain and recipient strain (*Escherichia coli* J53 azide resistant) were plated in a ratio of 1:5 on Luria Agar plates and incubated at 37 °C. Transconjugants were selected on two types of agar plates containing: (A) cefoxitin (10 mg/L) and sodium azide (100 mg/L) and (B) ciprofloxacin (0.06 mg/L) and sodium azide (100 mg/L), as recommended by earlier studies [21, 22]. Isolates which could not transfer their plasmid through conjugation were subjected to electro-transformation using *E. coli* DH10B as host cells. Transformants were selected in LA plates containing cefoxitin (5 mg/L). In one case where there was no colony on cefoxitin plate, transformants were selected on ampicillin (50 mg/L) agar plate. The transconjugants /transformants were screened for the presence of the *bla*_NDM_ gene, PMQRs (*aac(6′)-Ib-cr, qnrB* and *qnrS),* β-lactamases (*bla*_CTX-M_, *bla*_TEM_, *bla*_SHV_, *bla*_OXA-1_), and 16S rRNA methylases (*armA, rmtB, rmtC, rmtA, npmA, rmtD*) [23].

Plasmid DNA was isolated from wild-type and transconjugants/transformants by modified Kado and Liu plasmid isolation technique [24] and was sized by Quantity One® 1-D analysis software (Biorad) comparing with plasmids of *E. coli* V517 and *Shigella flexineri* YSH6000. Plasmid addiction systems *(pemKI, ccdAB, relBE, parDE, vagCD, hok–sok, pndCA, srnBC)* were investigated by PCR assays [25]. Plasmid types were also determined by PBRT kit (Diatheva srl, Cartoceto, Italy). Presence of class 1, class 2, and class 3 integrons was investigated [26].

The upstream and downstream regions of *bla*_NDM_ were amplified and sequenced with a series of primers which were designed previously [27].

### Statistics

Determination of significant differences between NDM-positive isolates and NDM-negative isolates and between organisms *Escherichia coli* and *Klebsiella pneumoniae* was calculated using the chi-square test of independence by comparing the variables. All statistical testing was two-tailed and all comparisons were unpaired. Statistical significance was defined as *P* ≤ 0.05.

## Results

### Isolates

Seventy-three isolates were identified as *Enterobacteriaceae* which included *Klebsiella pneumoniae* (75%, 55/73), *Escherichia coli* (22%, 16/73), *Enterobacter cloacae* (1%, 1/73) and *Enterobacter aerogenes* (1%, 1/73).

### Antibiotic susceptibility pattern

Ninety-seven percent (71/ 73) of the isolates were multidrug resistant (MDR) i.e. non-susceptible to three or more groups of antibiotics. Thirty isolates were resistant to 7 groups of antibiotics. Isolates were highly resistant to most of the antibiotics except meropenem and tigecycline, resistance was generally higher in *K. pneumoniae* than *E. coli.* Non-susceptibility to different antibiotics for all isolates is depicted in Table 1 and Additional file 1. *Enterobacter aerogenes* and *Enterobacter cloacae* were non-susceptible to piperacillin, cefotaxime, cefoxitin, ciprofloxacin, and aztreonam. Additionally, *Enterobacter aerogenes* was non-susceptible to meropenem and *Enterobacter cloacae* isolate was non-susceptible to gentamicin.

**Table 1:** Antibiotic susceptibility pattern of the studied isolates

Antibiotics	Non-susceptible isolates no. (%)	Non-susceptible *E. coli* isolates no.(%)	Non-susceptible *K. pneumoniae* isolates. no. *(*%*)*	*P* value *(E. coli / K.pn****.)***
Piperacillin	72 (99%)	15 (94%)	55 (100%)	0.5080
Cefotaxime	68 (93%)	13 (81%)	53 (96%)	0.1274
Cefoxitin	55 (75%)	9 (56%)	44 (80%)	0.1160
Aztreonam	65 (89%)	13 (81%)	50 (91%)	0.5311
Meropenem	36 (49%)	6 (38%)	29 (53%)	0.4306
Ciprofloxacin	66 (90%)	12 (75%)	52 (95%)	0.0670
Ofloxacin	58 (79%)	12 (75%)	46 (84%)	0.6753
Amikacin	55 (75%)	9 (56%)	45 (82%)	0.0756
Gentamicin	65 (89%)	11 (69%)	52 (95%)	0.0154
Sulfomethoxazole / trimethoprim	62 (85%)	11 (69%)	49 (89%)	0.1126
Tigecycline	16 (22%)	1 (6%)	15 (27%)	0.1523

Statistically significant *P* values are underlined

Since this study focuses on carbapenem and fluoroquinolone resistance, analysis of the isolates was carried out in terms of these two antibiotics separately. Forty-nine percent (36/73) of the total isolates were non-susceptible to meropenem and nearly all (97%, 35/36) meropenem non-susceptible isolates, were non-susceptible to ciprofloxacin, which included *E. coli* (*n* = 6)*, K. pneumoniae* (*n* = 29) and *Enterobacter aerogenes* (*n* = 1). Eighty-four percent (31/37) of the meropenem susceptible isolates were also non-susceptible to ciprofloxacin. Overall, ciprofloxacin non-susceptibility (90%) was higher than meropenem non-susceptibility (49%).

The range of MIC against meropenem in *E. coli* was 0.032 mg/L to > 32 mg/L and in *K. pneumoniae* was 0.023 mg/L to 32 mg/L. Whereas, MIC against ciprofloxacin in *E. coli* was 0.25 mg/L to > 32 mg/L and in *K. pneumoniae* was 0.064 mg/L to > 32 mg/L. The MIC of meropenem in *Enterobacter aerogenes* and *Enterobacter cloacae* were 4 mg/L and 0.047 mg/L respectively whereas MIC of ciprofloxacin were 4 mg/L and 15 mg/L. In *E. coli* isolates MIC50 and MIC 90 of meropenem were 0.125 mg/L and 24 mg/L respectively and in *K. pneumoniae* isolates MIC_50_ and MIC_90_ of meropenem were 1.5 mg/L and 10 mg/L respectively. In both organisms, MIC_50_ and MIC_90_ of ciprofloxacin were > 32 mg/L.

### Prevalence of various β-lactamases and PMQRs

Since *bla*_NDM-1_ genes can persist even in cells exhibiting very low-level resistance to meropenem [28], all isolates were screened for *bla*_NDM_ and other carbapenemases. Forty-seven percent (34/73) isolates were *bla*_NDM_-positive which included 6 *E. coli*, 27 *K. pneumoniae,* and 1 *Enterobacter aerogenes* isolate. No other metallo-β-lactamase was found. Sequencing of *bla*_NDM_ amplified product revealed that most of them were *bla*_NDM-1_, except 3 which were *bla*_**NDM-5**,_ *bla*_**NDM-7**_ and *bla*_**NDM-15**._ The *bla*_NDM**-15**_ was a novel variant and the sequence was submitted to GenBank (accession no. KP735848). Two isolates non-susceptible to meropenem, yet lacking *bla*_NDM_, possessed *bla*_OXA-48_. *bla*_KPC_ was not present in any of the isolates. β-lactamase genes *bla*_CTX-M_, *bla*_SHV_, *bla*_TEM_, *bla*_OXA-1_ were present in 66% (48/73), 49% (36/73), 42% (31/73) and 66% (48/73) isolates respectively (Table 2, Additional file 1).

**Table 2:** Distribution of resistance genes in studied organisms

Resistance Markers	Total (n = 73)	*E. coli* (*n* = 16)	*K. pneumoniae* (*n* = 55)	*Enterobacter sp. (n* = *2)*	*P value (E. coli/ K.pn.)*
*bla*_NDM_	34 (47%)	6 (38%)	27 (49%)	1	0.5938
PMQR (number)	59 (81%)	7 (44%)	50 (91%)	2	0.0001
*aac(6′)-Ib-cr*	52 (71%)	7 (44%)	44 (80%)	1	0.0117
*qnrB*	37 (51%)	0 (0%)	35 (64%)	2	0.0001
*qnrS*	2 (3%)	0 (0%)	2 (4%)	0	0.4391
*oqxAB*	55 (75%)	2 (13%)	53 (96%)	0	0.0001
*bla*_CTX-M_	48 (66%)	8 (50%)	38 (69%)	2	0.2671
*bla*_TEM_	31 (42%)	11 (69%)	20 (36%)	0	0.0442
*bla*_SHV_	36 (49%)	2 (13%)	34 (62%)	0	0.0014
*bla*_OXA_	48 (66%)	7 (44%)	39 (71%)	2	0.0883
*aac(6′)-Ib*	18 (25%)	0 (0%)	18 (33%)	0	0.0202

Statistically significant *P* values are underlined

Overall 81% (59/73) isolates were confirmed to carry at least one of the PMQRs which included 7 *E. coli* isolates, 50 *K. pneumoniae* isolates and both the *Enterobacter sp.* Overall, 40% (29/73) isolates co-harboured NDM and PMQRs*.* Among *qnr* genes, *qnrB* and *qnrS* were present in 51% (37/73) and 3% (2/73) of isolates respectively. Other *qnr* genes *qnrA, qnrC,* and *qnrD* were absent*.* All *qnrB* and *qnrS* genes found were *qnrB1* and *qnrS1* respectively. Seventy-one percent (52/73) isolates were positive for modifying enzyme coding *aac(6′)-Ib-cr* gene. Fourteen isolates carried both the *aac(6′)-Ib* and *aac(6′)-Ib-cr* alleles. None of the isolates carried plasmid-mediated efflux pump gene *qepA.* MDR family efflux pump genes *oqxA* and *oqxB* were found in 53 *K. pneumoniae* and 2 *E. coli* isolates. Prevalence of *aac(6′)-Ib-cr* in *K. pneumoniae* 80% (44/55) was significantly higher than *E. coli* 44% (7/16) (*P-value* 0.0117). In *K. pneumoniae* prevalence of *qnrB* and *qnrS* were 64% (35/55) and 4% (2/55) respectively but these genes were absent in *E. coli* (Table 2).

### Distribution of PMQRs in NDM-positive and NDM-negative isolates

An analysis of the distribution of PMQRs was carried out in NDM-positive and NDM-negative isolates. PMQR genes were highly abundant in both NDM-positive isolates (85%, 29/34) and NDM-negative isolates (77%, 30/39) (Table 3). Ninety-seven percent (33/34) NDM-positive isolates were non-susceptible to ciprofloxacin against 85% (33/39) of the NDM-negative isolates. Prevalence of *aac(6′)-Ib-cr* (82%,) and *qnrB* (56%) was higher in NDM-positive isolates than NDM-negative isolates (62 and 46% respectively). Of all isolates, *qnrS* was found only in 2 isolates which also possessed NDM (Table 3). Since *oqxA* and *oqxB* genes are mostly chromosomally located in *K. pneumoniae* [29], we have excluded this from the calculation of the total percentages of PMQRs.

**Table 3:** The difference between NDM-positive and NDM-negative isolates with respect to ciprofloxacin non-susceptibility and prevalence of PMQRs

Characteristics	NDM-positive isolates [*n* = 34]	NDM-negative isolates [*n* = 39]	*P* value
Ciprofloxacin nonsusceptibility	33 (97%)	33 (85%)	0.1607
Isolates carrying PMQR	29 (85%)	30 (77%)	0.5430
*aac(6′)-Ib-cr*	28 (82%)	24 (62%)	0.0890
*qnrB*	19 (56%)	18 (46%)	0.5521
*qnrS*	2 (6%)	0 (0%)	0.4139
*oqxAB*	27 (79%)	28 (72%)	0.6305

### Relatedness of the studied isolates based on PFGE patterns

**Fig. 1:**
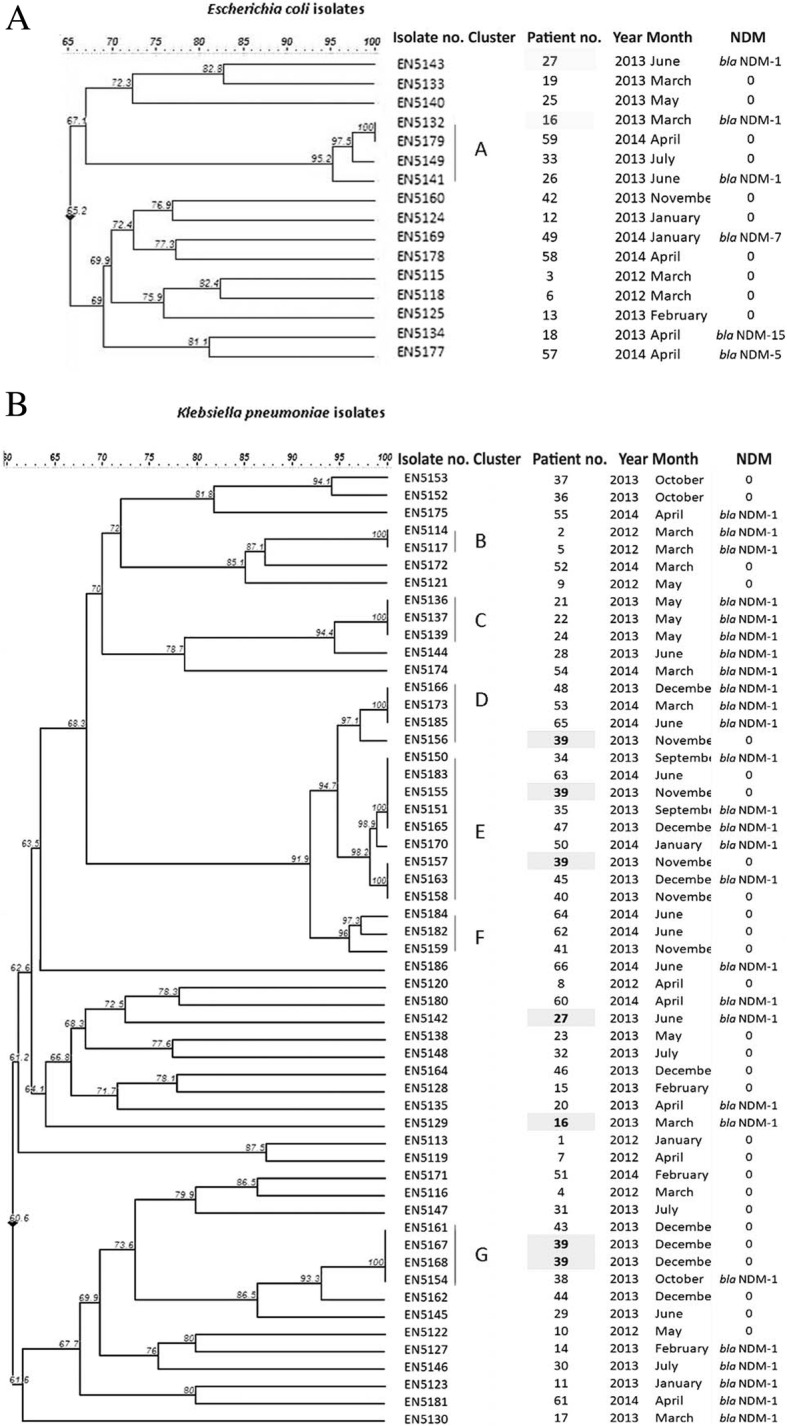
Genetic relatedness of (**a**) *E. coli* and (**b**) *K. pneumoniae* isolates. Analysis of PFGE of *Xba*I digestion pattern based on Dice’s similarity co-efficient and UPGMA (the position tolerance and optimization were set at 1.5 and 1.5% respectively). More than 95% similarity in PFGE band pattern was interpreted as indistinguishable

According to the cladogram, majority (12/16) of the *E. coli* isolates were diverse (Fig. 1a), except 4 isolates which were indistinguishable and grouped as cluster A. However, the cladogram of *K. pneumoniae* showed (Fig. 1b) that many isolates were indistinguishable. They were grouped into 6 clusters (cluster B – G). Cluster B, C, D, F, and G include 2–4 identical isolates. Cluster E was the largest cluster which included 9 isolates. The presence of a higher number of clonal isolates in *K. pneumoniae* may have contributed to the higher rate of fluoroquinolone non-susceptibility and prevalence of PMQRs in *K. pneumoniae* compared to *E. coli.*

### Detailed molecular characterization of NDM-possessing isolates with a focus on co-transfer of *bla*_NDM_ and PMQRs

#### Study of the mutations in GyrA and ParC in NDM-possessing isolates

**Fig. 2:**
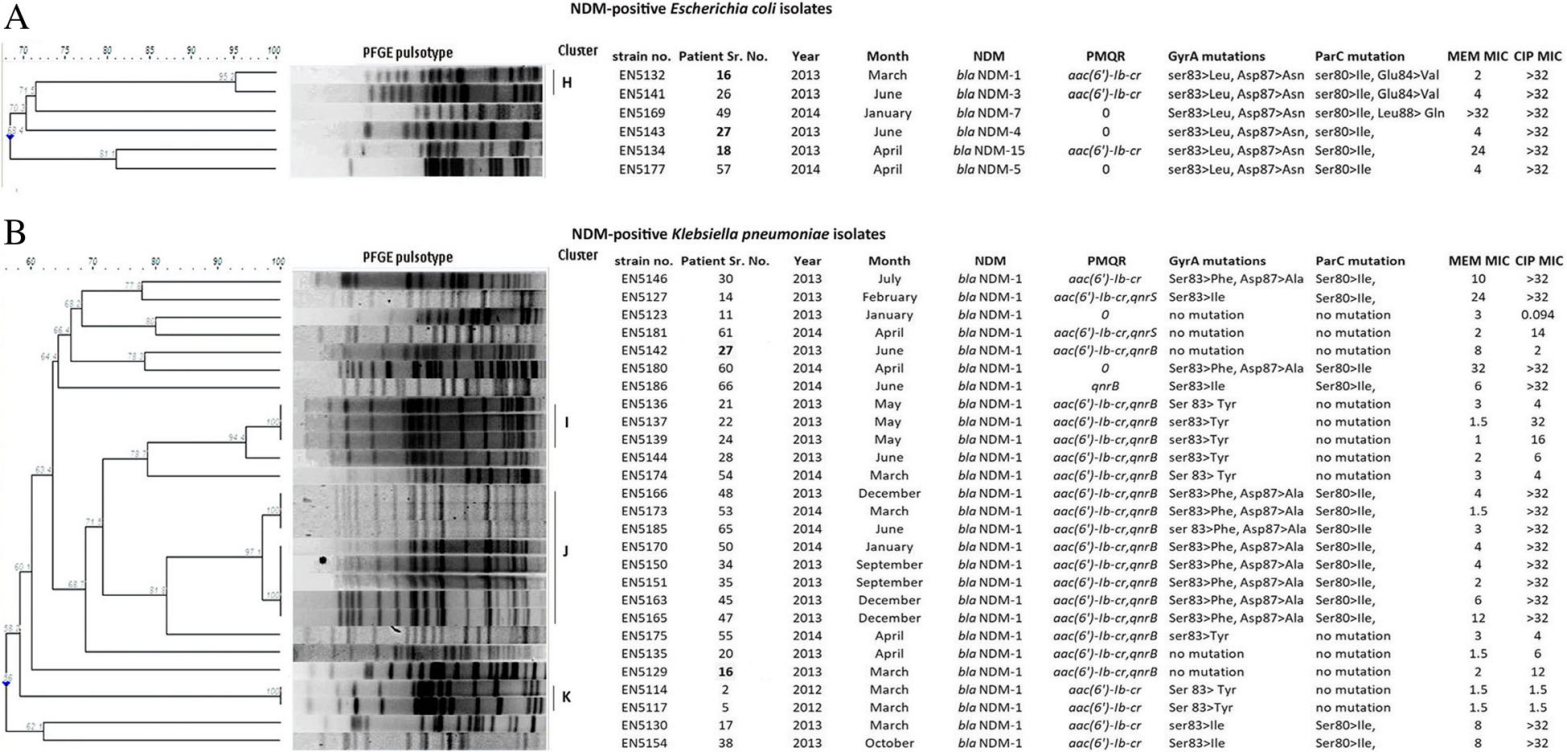
Genetic relatedness, the presence of PMQRs and chromosomal mutations and MIC values of meropenem and ciprofloxacin in NDM-positive (**a**) *E. coli* and (**b**) *K. pneumoniae* isolates. Analysis of PFGE of *Xba*I digestion pattern based on Dice’s similarity co-efficient and UPGMA (the position tolerance and optimization were set at 1.5 and 1.5% respectively). More than 95% similarity in PFGE band pattern interpreted as indistinguishable. MEM: meropenem, CIP: ciprofloxacin

Since fluoroquinolone resistance in *Enterobacteriaceae* results also from the accumulation of mutations primarily in DNA gyrase (GyrA*)* and then in topoisomerase IV (ParC*)*, sequences of the QRDR of NDM-positive isolates were studied for mutations in *gyrA* and *parC* genes. All 6 *E. coli* isolates carrying NDM had mutations in GyrA at codons 83 (Ser > Leu) and 87 (Asp>Asn) as well as in ParC at codon 80 (Ser > Ile). An additional mutation in ParC was present at codon 88 (Leu > Gln) in one isolate and at codon 84(Glu > Val) in two isolates which were clonally indistinguishable (EN5132, EN5141) (Fig. 2a).

*K. pneumoniae* isolates possessed varied mutations: Ser83Phe and Asp87Ala in GyrA along with Ser80Ile in ParC (*n* = 10); Ser83Ile mutation in GyrA and Ser80Ile ParC (*n* = 4) and Ser83Tyr mutation only in GyrA (*n* = 8). Five isolates had no mutation in the QRDR region (Fig. 2b). The *Enterobacter aerogenes* isolate possessed no mutation in GyrA or ParC.

In general, isolates accumulating mutations in both GyrA and ParC had higher MIC values (> 32 mg/L) than isolates possessing mutations in only GyrA (1.5–32 mg/L) (Fig. 2a and b).

Analysis of the interplay between chromosomal mutations and PMQRs and its effect on the MIC of ciprofloxacin is represented in Fig. 2b. Four *K. pneumoniae* isolates (EN5129, EN5135, EN5142, and EN5181) which had acquired PMQRs but lacked QRDR mutations were non-susceptible to ciprofloxacin according to CLSI criteria. Their MIC values were 12, 6, 2, and 14. Whereas one isolate (EN5123) which lacked both PMQRs and mutation in QRDR had a very low MIC (0.094 mg/L). *Enterobacter aerogens* [EN5131] also lacked the chromosomal mutations but carried PMQRs (*aac(6′)-Ib-cr* and *qnrB*) and MIC against ciprofloxacin was 4 mg/L. Overall, 82% NDM-positive isolates possessed GyrA and/or ParC mutation.

#### Transfer of resistance genes by conjugation / transformation assays and characterization of plasmids

Genetic transference of *bla*_NDM_ and PMQRs was studied by conjugation (*n* = 28) or transformation (*n* = 5) assays. Out of the 29 isolates co-harbouring PMQRs and *bla*NDM, 12 (41%) isolates including *E. coli* and *K. pneumoniae* co-transferred the resistance markers in large plasmids. *Aac(6′)-Ib-cr* co-transferred with *bla*_NDM_ in 18% (5/28) isolates which co-harboured the genes. Similarly, *qnrB* co-transferred with *bla*_NDM_ in 47% (9/19) cases. One of the two isolates co-harbouring *qnrS* transferred the gene along with *bla*_NDM._ One isolate [EN5129] possessed both *bla*_NDM_ and PMQRs but did not transfer the *bla*_NDM_ gene. Detailed molecular characteristics of these isolates and their transconjugants/transformants are presented in Tables 4 and 5. An analysis of the transconjugants of *E. coli* and *K. pneumoniae* is presented below separately.

**Table 4:** Genotypic characterization of NDM-positive isolates co-transferring *bla*_NDM-1_ and PMQRs

Strain no.	Organism	NDM	PMQR	Other Resistance genes	MEM MIC	CIP MIC	Plasmid size	Plasmid type	Plasmid addiction system	Integron
EN5132	*Escherichia coli*	*bla*_NDM-1_	*aac(6′)-Ib-cr*	*bla*_CTX-M, TEM, OXA_ *armA*	2	> 32	212, 6, 3	FIB,A/C,FIIK,FII,N	PemK, CcdAB, Hok-Sok	intI1
EN5132.T1	*E. coli* J53 Fox-azide	*bla*_NDM-1_	*aac(6′)-Ib-cr*	*bla*_CTX-M, TEM, OXA_ *armA*	2	0.047	212	A/C	–	intI1
EN5127	*Klebsiella pneumoniae*	*bla*_NDM-1_	*aac(6′)-Ib-cr, qnrS*	*aac(6′)-Ib, bla*_SHV,_ *rmtC*	24	> 32	210, 4,3	A/C,FIIK,N	–	intI1
EN5127.T1	*E. coli* J53 Fox-azide	*bla*_NDM-1_	*aac(6′)-Ib-cr*	*aac(6′)-Ib, rmtC*	2	0.016	210	N, A/C	–	IntI1
EN5127.T2	*E. coli* J53 Cip-Azide	*bla*_NDM-1_	*aac(6′)-Ib-cr*	*aac(6′)-Ib, rmtC*	6	0.5	210	N, A/C	–	IntI1
EN5150	*Klebsiella pneumoniae*	*bla*_NDM-1_	*aac(6′)-Ib-cr,qnrB*	*aac(6′)-Ib, bla*_CTX-M, SHV, OXA_	4	> 32	248	FIIK,	PemK	IntI1
EN5150.T1	*E. coli* J53 Fox-azide	*bla*_NDM-1_	*aac(6′)-Ib-cr,qnrB*	*aac(6′)-Ib bla*_CTX-M, OXA_	4	0.25	248	FIIK,	–	IntI1
EN5150.T2	*E. coli J53* Cip-Azide	*bla*_NDM-1_	*aac(6′)-Ib-cr,qnrB*	*aac(6′)-Ib bla*_CTX-M, OXA_	4	0.25	248	FIIK,		IntI1
EN5151	*Klebsiella pneumoniae*	*bla*_NDM-1_	*aac(6′)-Ib-cr,qnrB*	*aac(6′)-Ib bla*_CTX-M, SHV,OXA_	2	> 32	248, 128	FIIK,	–	IntI1
EN5151.T1	*E. coli* J53 Fox-azide	*bla*_NDM-1_	*qnrB*	*aac(6′)-Ib,*	1	0.064	248	FIIK,	–	IntI1
EN5151.T2	*E. coli* J53 Cip-Azide	*bla*_NDM-1_	*qnrB*	*aac(6′)-Ib*	0.5	0.047	248	FIIK,		IntI1
EN5163	*Klebsiella pneumoniae*	*bla*_NDM-1_	*aac(6′)-Ib-cr,qnrB*	*bla*_CTX-M, SHV, OXA,_ *aac(6′)-Ib*	6	> 32	266, 154	FIIK	–	IntI1
EN5163.T1	*E. coli* J53 Fox-azide	*bla*_NDM-1_	*qnrB*	*aac(6′)-Ib*	3	0.125	266	FIIK	–	IntI1
EN5163.T2	*E. coli* J53 Cip-Azide	*bla*_NDM-1_	*qnrB*	*aac(6′)-Ib*	2	0.125	266	FIIK		IntI1
EN5165	*Klebsiella pneumoniae*	*bla*_NDM-1_	*aac(6′)-Ib-cr,qnrB*	*aac(6′)-Ib bla*_CTX-M, SHV, OXA_	12	> 32	266, 154	FIIK	–	IntI1
EN5165.T1	*E. coli* J53 Fox-azide	*bla*_NDM-1_	*qnrB*	*aac(6′)-Ib*	0.38	0.75	266	FIIK		IntI1
EN5165.T2	*E. coli* J53 Cip-Azide	*bla*_NDM-1_	*qnrB*	*aac(6′)-Ib*	2	0.064	266	FIIK		IntI1
EN5166	*Klebsiella pneumoniae*	*bla*_NDM-1_	*aac(6′)-Ib-cr,qnrB*	*aac(6′)-Ib bla*_CTX-M, SHV, OXA_	4	> 32	266, 154	FIIK	–	IntI1
EN5166.T1	*E. coli* J53 Fox-azide	*bla*_NDM-1_	*qnrB*	*aac(6′)-Ib*	0.5	0.047	266	FIIK	–	IntI1
EN5166.T2	*E. coli* J53 Cip-Azide	*bla*_NDM-1_	*qnrB*	*bla*_CTX-M, OXA_ *aac(6′)-Ib*	3	0.064	266	FIIK		IntI1
EN5170	*Klebsiella pneumoniae*	*bla*_NDM-1_	*aac(6′)-Ib-cr, qnrB*	*aac(6′)-Ib, bla*_CTX-M, SHV, OXA_	4	> 32	260, 200	FIIK	–	IntI1
EN5170.T1	*E. coli* J53 Fox-azide	*bla*_NDM-1_	*qnrB*	*aac(6′)-Ib,*	0.5	0.064	260	FIIK	–	IntI1
EN5170.T2	*E. coli* J53 Cip-Azide	*bla*_NDM-1_	*qnrB*	*aac(6′)-Ib*	0.5	0.25	260	FIIK		IntI1
EN5173	*Klebsiella pneumoniae*	*bla*_NDM-1_	*aac(6′)-Ib-cr,qnrB*	*bla*_CTX-M, SHV, OXA_ *aac(6′)-Ib*	1.5	> 32	116, 90	FIIK,	–	IntI1
EN5173.T1	*E. coli* J53 Fox-azide	*bla*_NDM-1_	*qnrB*	*aac(6′)-Ib*	0.5	0.094	116	FIIK	–	IntI1
EN5173.T2	*E. coli* J53 Cip-Azide	*bla*_NDM-1_	*qnrB*	*aac(6′)-Ib*	1	0.047	116	FIIK		IntI1
EN5174	*Klebsiella pneumoniae*	*bla*_NDM-1_	*aac(6′)-Ib-cr,qnrB*	*bla*_CTX-M, SHV,OXA_ *, armA*	3	16	260, 7, 5, 4	FIIK, N, HIB-M	VagC/D	IntI1
EN5174.T1	*E. coli* J53 Fox-azide	*bla*_NDM-1_	*aac(6′)-Ib-cr,qnrB*	*bla*_CTX-M, SHV,OXA_, *armA*	3	6	260, 7, 5, 4	FIIK, N, HIB-M	Vagc/D	IntI1
EN5181	*Klebsiella pneumoniae*	*bla*_NDM-1_	*aac(6′)-Ib-cr,qnrS*	*bla*_CTX-M, SHV, TEM,_	2	14	220	FIIK, FII	PemK	–
EN5181.T1	*E. coli* J53 Fox-azide	*bla*_NDM-1_	*qnrS*	*bla*_CTX-M, TEM_	0.5	0.19	220	FIIK	–	–
EN5181.T2	*E. coli* J53 Cip-Azide	*bla*_NDM-1_	*qnrS*	*bla*_CTX-M, TEM_	0.75	0.19	220	FIIK	–	–
EN5185	*Klebsiella pneumoniae*	*bla*_NDM-1_	*aac(6′)-Ib-cr,qnrB*	*bla*_CTX-M, SHV, OXA_ *aac(6′)-Ib*	3	> 32	266, 154	FIIK	–	IntI1
EN5185.T1	*E. coli* J53 Fox-azide	*bla*_NDM-1_	*aac(6′)-Ib-cr,qnrB*	*aac(6′)-Ib*	0.5	0.032	266	FIIK	–	IntI1
EN5185.T2	*E. coli* J53 Cip-Azide	*bla*_NDM-1_	*aac(6′)-Ib-cr,qnrB*	*aac(6′)-Ib*	1.5	0.19	266	FIIK	-	IntI1

MEM: meropenem, CIP: ciprofloxacin, (-): absent or untypable (in case of plasmid types), *E. coli* J53 Fox-azide or ‘.T1’ : transconjugants selected in cefoxitin (10μg/ml)-sodium azide (100μg/ml), ), *E. coli* J53 Cip-Azide or ‘.T2’ : transconjugants selected in ciprofloxacin (0.06 μg/ml)-sodium azide (100 μg/ml)

**Table 5:** Genotypic characterization of NDM-positive isolates transferring only *bla*_NDM_ gene

Strain no.	Organism	NDM	PMQR	Other Resistance genes	MEM MIC	CIP MIC	Plasmid size in kb (approximately)	Plasmid type (Inc)	Plasmid addiction system	Integron
EN5134	*Escherichia coli*	*bla*_NDM-15_	*aac(6′)-Ib-cr*	*bla*_CTX-M, TEM, OXA_ *rmtB*	24	> 32	225, 158,4	FIA, FII, I1A	PemK, CcdAB, Hok-Sok, PndCA	intI1
EN5134.T1	*E. coli* J53 Fox-azide	*bla*_NDM-15_	*–*	*bla*_TEM,_ *rmtB*	2	0.012	121	FII	–	intI1
EN5141	*Escherichia coli*	*bla*_NDM-1_	*aac(6′)-Ib-cr*	*bla*_CTX-M, TEM, OXA,_ *armA*	4	> 32	246, 134, 6	FIB, HIB-M, FII	PemK, Hok-Sok	–
EN5141.T1	*E. coli* J53 Fox-azide	*bla*_NDM-1_	*–*	*bla*_TEM,_	1.5	0.008	246, 6	HIB-M	–	–
EN5143	*Escherichia coli*	*bla*_NDM-1_	*–*	*bla*_TEM_ *rmtB*	4	> 32	105,56, 9	FIA, I1_γ_, FII, I1a	PndC/A	intI1
EN5143.T1	*E. coli* J53 Fox-azide	*bla*_NDM-1_	*–*	*bla*_TEM_ *rmtB*	1.5	0.008	105	FIA, FII	–	intI1
EN5169	*Escherichia coli*	*bla*_NDM-7_	*–*	*bla*_TEM_ *rmtB*	> 32	> 32	200, 5, 2	I1A, FIA, FIB, FIIS, R, FII	PndCA, Hok-Sok, ccdAB, SrnBC, PemK	-
EN5169.T1	*E. coli* J53 Fox-azide	*bla*_NDM-7_	*–*	*bla*_TEM,_	1	0.012	200,5	I1A,	Pnd C/A	-
EN5177	*Escherichia coli*	*bla*_NDM-5_	*–*	*bla*_TEM_ *rmtB*	4	> 32	163,126,55,5, 2	FIB, FIIS	PemK	IntI1
EN5177.T1	*E. coli* J53 Fox-azide	*bla*_NDM-5_	*–*	*bla*_TEM_ *rmtB*	1	0.012	163,126	FIB, FIIS	–	IntI1
EN5114	*Klebsiella pneumoniae*	*bla*_NDM-1_	*aac(6′)-Ib-cr*	*bla*_CTX-M, SHV, OXA_ *armA*	1.5	1.5	248	HIB-M	PemK	intI1
EN5114.T1	*E. coli* J53 Fox-azide	*bla*_NDM-1_	*–*	*bla*_CTX-M_ *armA*	1.5	0.012	248,180	HIB-M	–	-
EN5117	*Klebsiella pneumoniae*	*bla*_NDM-1_	*aac(6′)-Ib-cr*	*bla*_CTX-M_ *armA*	1.5	1.5	248	HIB-M	PemK	intI1
EN5117.T1	*E. coli* J53 Fox-azide	*bla*_NDM-1_	*–*	*bla*_CTX-M, SHV, OXA_ *armA*	1	0.016	248	HIB-M	–	-
EN5123	*Klebsiella pneumoniae*	*bla*_NDM-1_	*–*	*bla*_CTX-M,_ *aac(6′)-Ib*	3	0.094	210, 20,7,5	FIA, R, FIIK	–	intI1
EN5123.T1	*E. coli* J53 Fox-azide	*bla*_NDM-1_	*–*	*bla*_CTX-M,_ *aac(6′)-Ib*	1	0.008	210,5	FIIK	–	-
EN5129	*Klebsiella pneumoniae*	*bla*_NDM-1_	*aac(6′)-Ib-cr,qnrB*	*bla*_CTX-M,_ *aac(6′)-Ib*	2	12	263,230,15.6,6.7,	FIIK, R	–	intI1
EN5129.T1F-A	*E. coli* DH10B	-	*aac(6′)-Ib-cr,qnrB*	*bla*_CTX-M_	0.023	0.19	230	FIIK	–	-
EN5130	*Klebsiella pneumoniae*	*bla*_NDM-1_	*aac(6′)-Ib-cr*	*bla*_CTX-M, OXA_	8	> 32	270,205,29,13, 8, 7, 6,5, 3, 2	FIIK, FIA, X2	–	intI1
EN5130.T1F	*E. coli* DH10B	*bla*_NDM-1_	*–*	*bla*_CTX-M, OXA_ *-*	2	< 0.002	270, 205, 29, 13, 8, 7,5, 3, 2	–	–	-
EN5135	*Klebsiella pneumoniae*	*bla*_NDM-1_	*aac(6′)-Ib-cr,qnrB*	*bla*_CTX-M, TEM, SHV, OXA_	1.5	16	230, 5, 4, 2, 0.1	FIIK, HIB-M	VagCD,	intI1
EN5135.T1	*E. coli* J53 Fox-azide	*bla*_NDM-1_	*–*	*bla*_CTX-M_	1.5	0.004	230	HIB-M	–	–
EN5136	*Klebsiella pneumoniae*	*bla*_NDM-1_	*aac(6′)-Ib-cr,qnrB*	*bla*_CTX-M, TEM, OXA_	3	4	340, 212, 9, 6, 4	FIIK, HIB-M	PemK, CcdAB, VagCD, Hok-Sok	intI1
EN5136.T1	*E. coli* J53 Fox-azide	*bla*_NDM-1_	*–*	*bla*_CTX-M, TEM_	0.75	0.012	340	HIB-M	–	intI1
EN5137	*Klebsiella pneumoniae*	*bla*_NDM-1_	*aac(6′)-Ib-cr,qnrB*	*bla*_CTX-M, TEM, OXA_	1.5	32	310,162, 8, 6, 4	FIIK, HIB-M	PemK, CcdAB, VagCD, Hok-Sok	intI1
EN5137.T1	*E. coli* J53 Fox-azide	*bla*_NDM-1_	*–*	*bla*_CTX-M, TEM_	0.75	0.012	310	HIB-M	–	-
EN5139	*Klebsiella pneumoniae*	*bla*_NDM-1_	*aac(6′)-Ib-cr,qnrB*	*bla*_CTX-M, TEM, OXA_	1	16	250,112,5, 4	FIIK, HIB-M	PemK, VagCD	intI1
EN5139.T1	*E. coli* J53 Fox-azide	*bla*_NDM-1_	*–*	*bla*_CTX-M, TEM,_	1.5	0.23	250	HIB-M	–	-
EN5142	*Klebsiella pneumoniae*	*bla*_NDM-1_	*aac(6′)-Ib-cr,qnrB*	*bla*_CTX-M, TEM, OXA_ *rmtC*	8	2	248,104	FIIK, FIB-M	–	intI1
EN5142.T1	*E. coli* J53 Fox-azide	*bla*_NDM-1_	*–*	- *rmtC*	1.5	0.008	248, 104	–	–	intI1
EN5144	*Klebsiella pneumoniae*	*bla*_NDM-1_	*aac(6′)-Ib-cr,qnrB*	*bla*_CTX-M, SHV, OXA_ *armA*	2	6	227, 52, 5, 4	HIB-M	–	-
EN5144.T1	*E. coli* J53 Fox-azide	*bla*_NDM-1_	*–*	*bla*_CTX-M, SHV, OXA_ *armA*	0.5	0.016	227	HIB-M	–	-
EN5146	*Klebsiella pneumoniae*	*bla*_NDM-1_	*aac(6′)-Ib-cr*	*bla*_CTX-M,_ *rmtC, aac(6′)-Ib*	10	> 32	192,164, 7	FIA, A/C, FIIK	–	-
EN5146.T1F	*E. coli* DH10B	*bla*_NDM-1_	*–*	*bla*_CTX-M,_ *aac(6′)-Ib*	6	< 0.002	164	A/C	–	-
EN5154	*Klebsiella pneumoniae*	*bla*_NDM-1_	*aac(6′)-Ib-cr*	*bla*_CTX-M_ *armA*	8	> 32	241, 201, 34, 29,8, 6, 4,3	FIIS, R, FIIK	PemK, VagCD	IntI1
EN5154.T1F	*E. coli* DH10B	*bla*_NDM-1_	*–*	*bla*_CTX-M,_	6	0.012	240, 4	FIIS	–	IntI1
EN5175	*Klebsiella pneumoniae*	*bla*_NDM-1_	*aac(6′)-Ib-cr, qnrB*	*bla*_CTX-M, TEM, OXA_ *armA*	3	4	123, 88,7,5,3	N, FIIK, HIB-M	PemK, VagCD	IntI1
EN5175.T1	*E. coli* J53 Fox-azide	*bla*_NDM-1_	*–*	*bla*_CTX-M, SHV, OXA_ *armA*	1.5	0.012	123	HIB-M	-	-
EN5175.T2	*E. coli* J53 Cip-Azide	–	*qnrB*	*armA*	0.023	0.125	96, 50	N, FIIK	VagCD	-
EN5180	*Klebsiella pneumoniae*	*bla*_NDM-1_	*–*	*armA,rmtC, bla*_TEM_ *aac(6′)-Ib*	32	> 32	293,208,5,4	A/C, FIIK, FIB-M, HIB-M	VagCD	IntI1
EN5180.T1	*E. coli* J53 Fox-azide	*bla*_NDM-1_	*–*	*bla*_TEM_ *rmtC*	1.5	0.016	208	A/C		IntI1
EN5186	*Klebsiella pneumoniae*	*bla*_NDM-1_	*qnrB*	*armA, rmtC*	6	> 32	235,188,47,5, 3,	FII	–	IntI1,IntI2
EN5186.T1	*E. coli* J53 Fox-azide	*bla*_NDM-1_	*–*	*bla*_CTX-M, TEM_ *rmtC*	1.5	0.008	188	-	-	IntI1
EN5131	*Enterobacter aerogenosa*	*bla*_NDM-1_	*aac(6′)-Ib-cr,qnrB*	*bla*_CTX-M, OXA,_ *rmtC*	4	4	205,27,17,7,5	–	ccdA/B, hok -sok	intI1
EN5131.T1	*E. coli* DH10B	*bla*_NDM-1_	*–*	*bla*_CTX-M,_ *rmtC*	2	< 0.002	205,27,7	–	–	

MEM: meropenem, CIP: ciprofloxacin, (-) : absent or untypable (in case of plasmid types), *E. coli* J53 Fox-azide or ‘.T1’ : transconjugants selected in cefoxitin (10μg/ml)-sodium azide (100μg/ml), *E. coli* DH10B or ‘.TF’: Transformants selected in cefoxitin (5μg/ml) , ‘.TF-A : Transformants selected in ampicillin (50μg/ml), *E. coli* J53 Cip-Azide or ‘.T2’ : transconjugants selected in ciprofloxacin (0.06 μg/ml)-sodium azide (100 μg/ml)

**Fig. 3:**
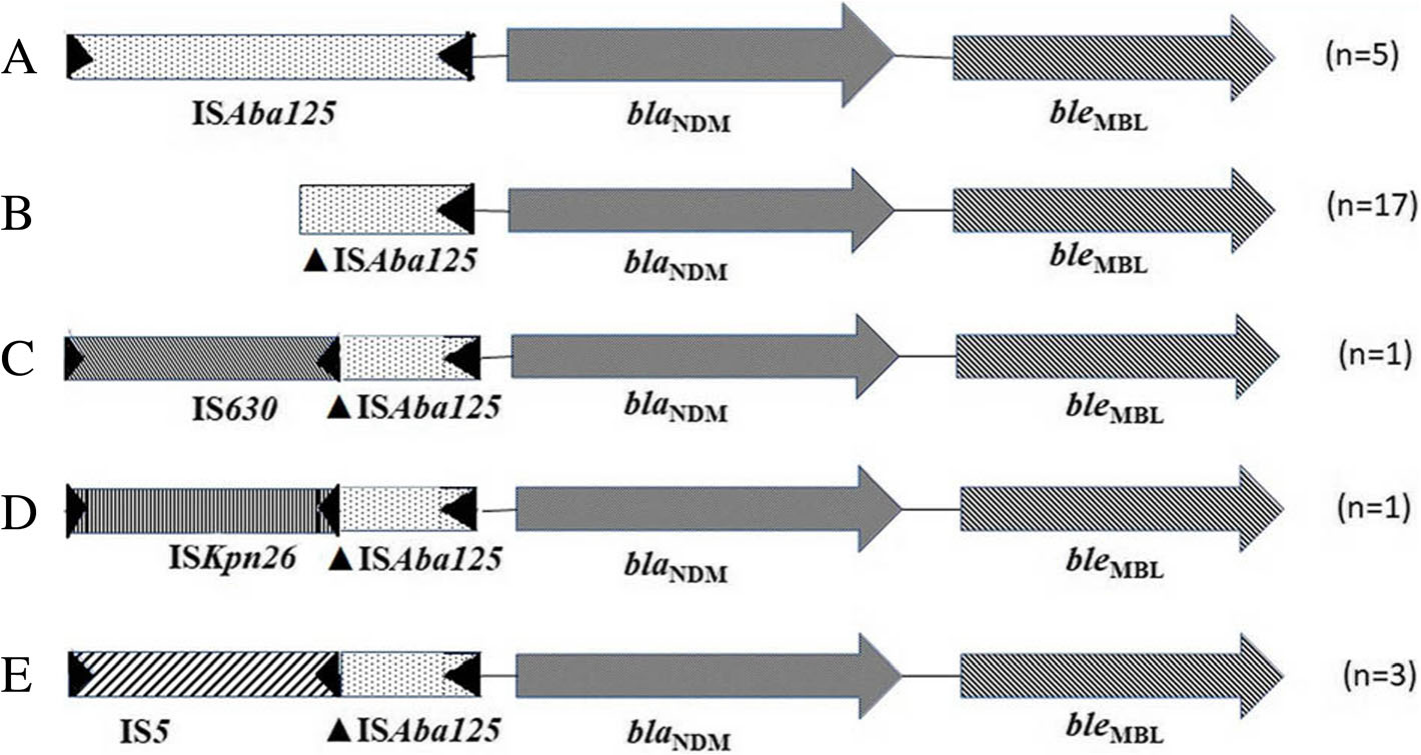
Upstream and downstream regions of the *bla*_NDM_ gene. Structure **a** was present in 5 isolates, structure **b** was present in 17 isolates, structure **c** and **d** were present in one isolate each and structure **e** was present in 3 isolates

All *bla*_NDM_-positive *E. coli* isolates (*n* = 6) carried multiple plasmids and were able to conjugally transfer their plasmid(s) carrying *bla*_NDM_ in selective plates containing cefoxitin (10 mg/L) and sodium azide (100 mg/L) but no transconjugants in ciprofloxacin (0.06 mg/L) and sodium azide (100 mg/L) plates. Among these, 3 possessed *aac(6′)-Ib-cr* but only in one case this gene co-transferred with *bla*_NDM_ in a single large 212 kb IncA/C type plasmid which also carried *IntI1* and various other resistance genes (*bla*_CTX-M,_ *bla*_TEM,_ *bla*_OXA,_ *armA*). In other *bla*_NDM-_positive *E. coli* isolates that did not possess PMQRs, *bla*_NDM_-harbouring plasmids were of varied replicon types such as IncFII, IncFIIS, IncHIB-M, IncI1A, IncF1A, and IncFIB. Study of the upstream region of *bla*_NDM_ revealed that 4 carried the complete IS*Aba125* and 2 carried a truncated version of it. One isolate [EN5169] possessed IS5 element followed by a truncated IS*Aba125* in the upstream region. *ble*_MBL_ was present in the downstream region of *bla*_NDM_ in all *E. coli* isolates (Fig. 3).

Twenty-six of 27 *bla*_NDM-1_-positive *K. pneumoniae* successfully transferred this gene either through conjugation (*n* = 22) or transformation (*n* = 4). Ninety-three percent (25/27) *K. pneumoniae* isolates co-harboured *bla*_NDM_ and at least one of the PMQRs. Among these, 11 *K. pneumoniae* yielded transconjugants co-harbouring *bla*_NDM_ and PMQRs. On analysis of the transconjugants, it was revealed that 10 of these isolates co-transferred the *bla*_NDM-1_ and PMQR genes in single large plasmids of IncFIIK, IncA/C and IncN type. It was noted that 8 of the 10 isolates co-transferring the genes on an IncFIIK plasmid were clonal (indistinguishable PFGE pattern) and this particular clone was isolated from the neonates between 2013 September to 2014 June (Table 4, Fig. 2b). Isolate EN5174 transferred both *bla*_NDM-1_ and PMQRs but multiple plasmids were isolated from the transconjugant. Hence, it was hard to determine whether PMQRs co-transferred with *bla*_NDM-1_ in a single plasmid or not. However, one isolate (EN5175) yielded different transconjugants on cefoxitin-sodium azide and ciprofloxacin-sodium azide plates. EN5175.T1 (selected on cefoxitin-sodium azide plate) harboured *bla*_NDM-1_ in an IncHIB-M plasmid whereas EN5175.T2 (selected on ciprofloxacin-sodium azide plate) harboured *qnrB* in IncFIIK or IncN plasmid. This showed that *bla*_NDM_ and *qnrB* were carried on different plasmids. Rest of the *K. pneumoniae* isolates (*n* = 14) only transferred *bla*_NDM_ in plasmids of type IncHIB-M (*n* = 8), IncA/C (*n* = 2), IncFIIK (*n* = 1) and untypable (*n* = 3). One *K. pneumoniae* did not transfer *bla*_NDM_ via conjugation or transformation.

Out of 27 *K. pneumoniae* isolates, 20 isolates had IS*Aba125* upstream *bla*_NDM-1,_ either complete (1/16) or truncated (15/16). Isolate EN5181 possessed IS*630* transposase, isolate EN5127 possessed IS*Kpn26* transposase, and isolates EN5135 and EN5174 possessed IS5 element followed by a truncated IS*Aba125* upstream *bla*_NDM-1_. The upstream region of 7 isolates could not be determined. All of the *K. pneumoniae* isolates had a *ble*_MBL_ gene which confers resistance to bleomycin in the downstream region of the *bla*_NDM_ gene (Fig. 3).

The *Enterobacter aerogenes* isolate acquired PMQRs but they were not transferred along with *bla*_NDM-1_ through transformation. This isolate carried a truncated IS*Aba125* in the upstream region and *ble*_MBL_gene in the downstream region. Various other resistance determinants (β-lactamases and 16 rRNA methylases) were transferred along with *bla*_NDM_ in all the organisms studied (Table 4).

Among the plasmid addiction systems found (*pndC/A, pemKI, ccdA/B, hok-sok, srnB/C, vagC/D,*) only *pndC/A* was present in a plasmid which carried *bla*_NDM-1_ in one case.

#### Clonality of NDM-possessing *E. coli* and *K. pneumoniae* isolates

NDM-possessing *E. coli* isolates (*n* = 6) were predominantly diverse except for 2 isolates which were indistinguishable [cluster H (EN5132, EN5141] (Fig. 2a). However, in case of *K. pneumoniae,* 3 clonal clusters [cluster I (EN5136, EN5137, EN5139), cluster J (EN5150, EN5151, EN5163, EN5165, EN5166, EN5170, EN5173, EN5185) and cluster K (EN5114, EN5117)] were identified and the rest were diverse. (Fig. 2b). Many identical isolates expressed different genotypic characteristics (Fig. 2, Tables 4 and 5).

## Discussion

The spread of antimicrobial resistance is primarily caused by the dissemination of large plasmids carrying multiple antibiotic resistance genes [6]. Antibiotic-resistant genes, such as *bla*_NDM-1_, are plasmid mediated and often co-harboured with different antibiotic resistance markers such as ESBL genes, aminoglycoside resistance markers and PMQRs [5]. PMQRs do not confer high-level resistance to fluoroquinolones, however, their presence in clinical isolates is of concern as it increases the risk of selecting mutations in gyrase and topoisomerase genes which results in high-level resistance [3]. With the increasing use of fluoroquinolones both in hospital settings and the community, PMQRs can be a palpable threat. In addition to this is the escalating presence of genes such as *bla*_NDM-1_ which can facilitate the spread of other plasmid-mediated genes as they may be present in the same plasmid or integrons. To the best of our knowledge, this is the first study which compares NDM-positive and NDM-negative *Enterobacteriaceae* isolates with respect to fluoroquinolone non-susceptibility and prevalence of PMQRs.

In the studied isolates, fluoroquinolone non-susceptibility was very high (90%). Other studies from India also show a very high rate of non-susceptibility to ciprofloxacin [30, 31]. A recent report from India shows that ciprofloxacin resistance was 15% at Day 1 and 38% in Day 60 in the gut flora of antibiotic naïve and exclusively breastfed neonates [32]. For treatment of neonatal infections, fluoroquinolones are used only as salvage therapy [7]. The high prevalence of fluoroquinolone resistance observed in the study is probably a reflection of the high usage of fluoroquinolones to treat other infections such as urinary tract infections (UTI) [32], as this drug used to be sold in India over the counter without prescription before 2014 [33]. It is also known that the mother’s vaginal flora may be a cause of sepsis (particularly early onset, the onset of sepsis within 72 h of birth) and mothers may be already harbouring such resistant organisms [34].

Forty-seven percent (34/73) of the isolates were NDM-positive. Majority of these possessed *bla*_NDM-1_ but isolates harbouring *bla*_NDM-5,_ *bla*_NDM-7,_ and a novel variant *bla*_NDM-15_ were also detected. The prevalence of *bla*_NDM-1_ is high in India [35, 36] and *bla*_NDM_ variants have also been reported [37].

NDM-positive isolates exhibited a higher percentage (97%) of non-susceptibility towards ciprofloxacin than NDM-negative (85%) but the difference was not statistically significant. In this study, a significant number of isolates (81%) carried at least one of the PMQRs. Analysis of the data also revealed that the prevalence of *aac(6′)-Ib-cr* was highest (71%) followed by *qnrB* (51%) and *qnrS* (3%). Earlier studies also support that *aac(6′)-Ib-cr* is the most prevalent PMQR in India [30, 38]. Although there are currently 81 variants of *qnrB* and 14 variants of *qnrS* according to https://www.ncbi.nlm.nih.gov/bioproject/PRJNA313047 [39], we have exclusively found only *qnrB1* and *qnrS1.* The prevalence of *aac(6′)-Ib-cr* was significantly higher in *K. pneumoniae* than *E. coli. qnrB* and *qnrS* were absent in *E. coli.* The higher prevalence of PMQRs in *K. pneumoniae* compared to *E. coli* can be the result of the presence of more clonal isolates of *K. pneumoniae* than *E. coli.* The prevalence of OqxAB was quite high as they are mostly chromosomally located in *K. pneumoniae* [29].

Co-occurrence of PMQRs and *bla*_NDM_ were reported in many earlier studies [5, 6, 21]. In this study, 40% (29/73) isolates co-harboured NDM and PMQRs. Although the prevalence of *aac(6′)-Ib-cr*, *qnrB,* and *qnrS* were generally higher in NDM-positive isolates than NDM-negative isolates the difference was not statistically significant. Hence, probably the spread of PMQRs is not dependent on the *bla*_NDM_ spread. The higher prevalence of PMQRs (81%) per se in comparison to NDM (47%) is also indicative of this. The occurrence of PMQRs along with β-lactamases has also been reported in several studies [6, 40]. It is to be noted that β-lactamases are highly prevalent in the study isolates and could have contributed to the spread of PMQRs.

Co-transfer of PMQRs along with *bla*_NDM_ in single large plasmids co-harbouring many other resistance genes have been shown in other studies [6, 21, 27]. The transfer of *bla*_NDM_ along with *qnrB, qnrS, aac(6′)-Ib-cr* and various other resistance markers (16S rRNA methylases and other β-lactamases genes) were studied. This study showed that of the 29 isolates which co-harboured NDM and PMQRs, only 12 isolates showed co-transmission of these genes which indicates that not all isolates possessing PMQRs co-transferred the gene with *bla*_NDM_ because of their probable location on different plasmids.

Worldwide studies on the plasmid types show that IncFII, IncN, IncL/M, IncHIB-M/IncFIB-M, IncA/C, and untypable plasmids carry *bla*NDM [21]. PMQRs are associated with IncN, IncL/M, IncFII, IncHI1, IncI1, IncR, colE type plasmids [41]. In this study, we have found that in *K. pneumoniae,* plasmids carrying both *bla*_NDM_ and PMQRs were of replicon type IncFIIK followed by IncA/C and IncN. IncF group plasmids are highly conjugative and are widely distributed in *Enterobacteriaceae* [41] and presence of any gene in this group of plasmids will only escalate its spread to other organisms. However, plasmid type IncHIB-M or an untypable plasmid was mostly associated with plasmids carrying *bla*_NDM_ but not any of the PMQRs. However, in *E. coli,* there were varied plasmid types, no particular type of plasmid predominated.

Fluoroquinolone resistance in *Enterobacteriaceae* is also caused by the accumulation of mutations, primarily in DNA gyrase (GyrA*),* and then in topoisomerase IV [3]. In our study, most NDM-positive isolates exhibited mutations in the QRDR region of GyrA and ParC. All of these mutations were reported earlier in various studies [42]. Four *K. pneumoniae isolate* and one *Enterobacter cloacae* carried PMQRs but lacked mutations in the QRDR region of GyrA and ParC, yet the isolates exhibited non-susceptible MIC values against ciprofloxacin. This indirectly points to the well-studied phenomenon that in the absence of chromosomal mutations PMQRs plays an important role in increasing the MIC against ciprofloxacin, thus providing an opportunity to the bacteria to generate chromosomal mutation [3].

## Conclusion

This study indicates that fluoroquinolone resistance is high in neonatal septicaemic isolates. PMQRs are highly prevalent, *aac(6′)-Ib-cr* and *qnrB* are predominant. Carbapenem resistance in the same set of isolates is primarily due to *bla*_NDM-1._ However, we infer that the spread of PMQRs is independent of the spread of *bla*_NDM-1_ as the prevalence of PMQRs in non-NDM isolates were nearly similar to the NDM isolates. The possibility of indiscriminate fluoroquinolone use in escalating the spread of *bla*_NDM-1_ cannot be ruled out. Co-occurrence of PMQRs with *bla*_NDM_ in an isolate does not necessarily result in co-transfer of the resistance genes due to their presence mostly in different plasmids. However, the presence of genes such as *bla*_NDM-1_and PMQRs shows that the window for treatment options are gradually decreasing and transmissible genes are a threat.

## Additional file

Additional file 1: Detailed information of all studied *Enterobacteriaceae* : MIC values of meropenem and cirpfloxacin, antibiotic susceptibility pattern and distribution of different resistance genes. (XLSX 28 kb)

## Abbreviations

NDM: New Delhi metallo-β-lactamase
PBRT: PCR-based replicon typing
PFGE: Pulsed-field gel electrophoresis
PMQR: Plasmid-mediated quinolone resistance
QRDR: Quinolone resistance determining region
